# Extracorporeal membrane oxygenation for life-threatening asthma refractory to mechanical ventilation: analysis of the Extracorporeal Life Support Organization registry

**DOI:** 10.1186/s13054-017-1886-8

**Published:** 2017-12-06

**Authors:** Hye Ju Yeo, Dohyung Kim, Doosoo Jeon, Yun Seong Kim, Peter Rycus, Woo Hyun Cho

**Affiliations:** 1 Department of Pulmonology and Critical Care Medicine, Pusan National University Yangsan Hospital, Geumo-ro 20, Beomeo-ri, Mulgeum-eup, Yangsan-si, Gyeongsangnam-do 50612 Republic of Korea; 2 Research Institute for Convergence of Biomedical Science and Technology Pusan National University Yangsan Hospital, Yangsan, Republic of Korea; 3 Department of Thoracic and Cardiovascular Surgery, Pusan National University Yangsan Hospital, Yangsan, Republic of Korea; 4 Extracorporeal Life Support Organization (ELSO), Ann Arbor, MI USA

**Keywords:** Extracorporeal Life Support Organization, Extracorporeal membrane oxygenation, Outcomes, Near-fatal asthma

## Abstract

**Background:**

The use of extracorporeal membrane oxygenation (ECMO) in cases of near-fatal asthma (NFA) has increased, but the benefits and potential complications of this therapy have yet to be fully investigated.

**Methods:**

Cases were extracted from the Extracorporeal Life Support Organization Registry between March 1992 and March 2016. All patients with a diagnosis of asthma (according to the International Classification of Diseases 9th edition), who also received ECMO, were extracted. Exclusion criteria included patients who underwent multiple courses of ECMO; those who received ECMO for cardiopulmonary resuscitation or cardiac dysfunction; and those with another primary diagnosis, such as sepsis. We analyzed survival to hospital discharge, complications, and clinical factors associated with in-hospital mortality, in patients with severe life-threatening NFA requiring ECMO support.

**Results:**

In total 272 patients were included. The mean time spent on ECMO was 176.4 hours. Ventilator settings, including rate, fraction of inspired oxygen (FiO_2_), peak inspiratory pressure (PIP), and mean airway pressure, significantly improved after ECMO initiation (rate (breaths/min), 19.0 vs. 11.3, *p* < 0.001; FiO_2_ (%), 81.2 vs. 48.8, *p* < 0.001; PIP (cmH_2_O), 38.2 vs. 25.0, *p* < 0.001; mean airway pressure (cmH_2_O): 21.4 vs. 14.2, *p* < 0.001). In particular, driving pressure was significantly decreased after ECMO support (29.5 vs. 16.8 cmH_2_O, *p* < 0.001). The weaning success rate was 86.7%, and the rate of survival to hospital discharge was 83.5%. The total complication rate was 65.1%, with hemorrhagic complications being the most common (28.3%). Other complications included renal (26.8%), cardiovascular (26.1%), mechanical (24.6%), metabolic (22.4%), infection (16.5%), neurologic (4.8%), and limb ischemia (2.6%). Of the hemorrhagic complications, cannulation site hemorrhage was the most common (13.6%). Using multivariate logistic regression analysis, it was found that hemorrhage was associated with increased in-hospital mortality (odds ratio, 2.97; 95% confidence interval, 1.07–8.24; *p* = 0.036). Hemorrhage-induced death occurred in four patients (1.5%). The most common reason for death was organ failure (37.8%).

**Conclusions:**

ECMO can provide adequate gas exchange and prevent lung injury induced by mechanical ventilation, and may be an effective bridging strategy to avoid aggressive ventilation in refractory NFA. However, careful management is required to avoid complications.

**Electronic supplementary material:**

The online version of this article (doi:10.1186/s13054-017-1886-8) contains supplementary material, which is available to authorized users.

## Background

Near-fatal asthma (NFA) is a life-threatening condition caused by acute respiratory failure, and is the most severe clinical presentation of asthma [1, 2]. Although there are no specific diagnostic criteria for NFA, it is characterized by cardiorespiratory arrest, hypercapnia, acidemia, and the need for intubation and mechanical ventilation [3]. NFA can progress to fatal asthma, and the mortality rate varies widely across countries. In 2015, there were 3615 deaths from asthma in the USA [4]. Despite recent advances in treatment, the mortality rate for asthma has not changed significantly. It may be that much of the current asthma mortality is due to unpredictable deaths in the context of NFA [5]. Globally, nearly 30% of NFA cases result in significant morbidity and mortality [4–10]. Traditionally, protective mechanical ventilation with rescue therapy, including neuromuscular blockade, has been the mainstay treatment for NFA. However, technical advances in extracorporeal membrane oxygenation (ECMO) have made it a promising alternative therapy in the treatment of life-threatening NFA [11–13].

ECMO can provide adequate gas exchange during acute respiratory failure, and can help prevent lung injury induced by aggressive mechanical ventilation. Using data from the multicenter Extracorporeal Life Support Organization (ELSO) Registry, a previous study showed that ECMO support improved survival in patients with *status asthmaticus* to a greater degree than in other respiratory conditions [11]. However, this study included a small number of cases. The low incidence of NFA may preclude large-scale, prospective randomized trials on the effectiveness of ECMO in this context. Nevertheless, an increasing number of reports suggest ECMO may be an effective rescue therapy for NFA [12, 13].

Despite the potential benefits of ECMO in NFA, concerns remain about complications. In general, complications are the primary factor limiting the effectiveness of ECMO in many diseases. Evidence supporting ECMO use in NFA is lacking, and questions about the safety and effectiveness of ECMO in NFA remain unanswered. In this study, we reviewed data from the ELSO Registry collected between March 1992 and March 2016. Our aim was to investigate survival to hospital discharge, complications, and clinical factors associated with in-hospital mortality, in patients with severe life-threatening NFA requiring ECMO support.

## Methods

The ELSO Registry is a worldwide voluntary registry. Currently, data are reported from 350 international ECMO centers. Data are collected using a standardized form, and include patient demographics, pre-ECMO ventilator settings, pre-ECMO arterial blood gas analysis (ABGA), post-ECMO ventilator settings, ECMO complications, and survival to hospital discharge [14]. Driving pressure (ΔP) was calculated as ventilator-measured plateau pressure minus applied positive end-expiratory pressure (PEEP). Diagnostic information is reported using the International Classification of Diseases 9th edition (ICD-9) codes, and indications for ECMO application are categorized as cardiac, respiratory, or extracorporeal cardiopulmonary resuscitation (ECPR). The study was approved by the Institutional Review Board (IRB) of Pusan National University Yangsan Hospital (IRB no.05-2017-066). Informed consent was not sought due to the retrospective nature of the study.

### Patient cohorts

**Fig. 1:**
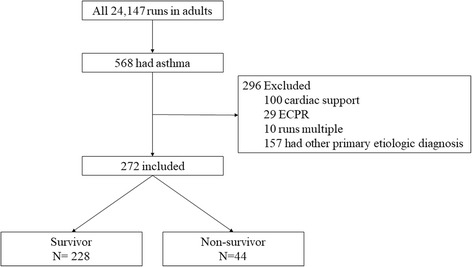
Patient inclusion in the study. In total, 272 patients were included in the study. ECPR, extracorporeal cardiopulmonary resuscitation

Between March 1992 and March 2016, 24,147 adults aged over 18 years were registered in the ELSO database. All cases of ECMO use in the context of an ICD-9 diagnosis of asthma were extracted (*N* = 568). Patients receiving ECMO support for ECPR or cardiac dysfunction were excluded according to the support type (ECPR, cardiac, or respiratory), and multiple ECMO courses were excluded. To discriminate between asthma and other non-asthmatic respiratory ECMO cases, patients with other primary diagnoses were also excluded. For example, a patient with asthma who received ECMO for sepsis was excluded, as sepsis was the primary indication for therapy. In total, 272 patients were included in the NFA group (Fig. 1).

### Statistical analysis

Data were reported as mean values with standard deviations for normally distributed data, or frequencies with proportions for categorical data. We compared the demographics, pre-ECMO and post-ECMO values, and ECMO complications between survivors and non-survivors among patients with NFA. Student’s *t* test was used for continuous data and the Fisher’s exact or Pearson’s chi-square test was used for categorical data. Pre-ECMO and post-ECMO ventilator settings were compared using the paired *t* test.

Binary logistic regression analysis was used to identify predictors of in-hospital mortality. Significant and borderline values (≤0.05) were entered into stepwise backward multivariable logistic regression, including age as the continuous covariate, to estimate factors associated with mortality prior to hospital discharge. Two-way interaction terms were tested between the remaining significant variables. A *p* value <0.05 was considered significant. Age, pre-ECMO ventilator settings, pre-ECMO ABGA, post-ECMO ventilator settings, and ECMO complications were used as predictors.

## Results

### Baseline patient characteristics

Among the 24,147 adult patients in the ELSO registry, 568 patients had a diagnosis of asthma according to the ICD-9 code (2.4%). Of these, 272 patients met the criteria for inclusion in the study. The use of ECMO for NFA in adults has been growing rapidly since 1992 (Additional file 1). The baseline clinical characteristics of the patients are presented in Table 1. Mean age was 36.2 years (range, 18–83 years). Mean body weight was 88.0 kg and. 39.7% of the patients were men. Pre-ECMO ABGA showed hypercapnic acidosis with mean pH of 7.1, mean partial pressure of carbon dioxide of 80.5 mmHg, and mean partial pressure of arterial oxygen/mean fraction of inspired oxygen (FiO_2_) ratio of 153.7. The mean values of the ventilator settings were relatively high. Prior to ECMO initiation, the mean peak inspiratory pressure (PIP) was 38.2 cmH_2_O; mean airway pressure was 21.4 cmH_2_O; mean PEEP was 8.3 cmH_2_O; mean FiO_2_ was 81.2%; and mean driving pressure was 29.2 cmH_2_O.

**Table 1:** Baseline patient characteristics

Variables	Total *N* = 272	Survivor *N* = 227	Non-survivor *N* = 45	*p*
Age	36.2 ± 13.4	34.7 ± 12.5	43.4 ± 15.8	0.001
Male, *N* (%)	108 (39.7)	93 (40.9)	15 (33.3)	0.356
Weight, kg	88.0 ± 29.1	87.5 ± 28.9	90.9 ± 30.4	0.501
Pre-ECMO blood gases^a^				
pH	7.1 ± 0.2	7.1 ± 0.2	7.2 ± 0.2	0.045
PCO_2_, mmHg	80.5 ± 50.6	81.1 ± 51.6	77.2 ± 45.5	0.638
PO_2_, mm Hg	109.6 ± 113.9	113.0 ± 115.5	91.3 ± 104.2	0.289
HCO_3_^-^, mmol/L	29.0 ± 9.4	29.2 ± 9.4	27.8 ± 9.6	0.402
SaO_2_, %	91.2 ± 10.1	92.3 ± 7.4	85.2 ± 17.3	0.030
Pre-ECMO ventilator setting				
Rate, breaths/min	19.0 ± 7.7	18.6 ± 7.4	21.4 ± 9.4	0.129
FiO_2_, %	81.2 ± 23.0	80.2 ± 23.2	87.2 ± 21.1	0.105
PIP, cmH_2_O	38.2 ± 11.1	38.1 ± 11.4	38.7 ± 9.1	0.798
PEEP, cmH_2_O	8.3 ± 5.9	7.8 ± 5.9	11.5 ± 5.1	0.002
MAP, cmH_2_O	21.4 ± 14.2	21.4 ± 15.1	21.3 ± 7.8	0.980
Driving pressure, cmH_2_O	29.5 ± 12.9	29.9 ± 13.4	27.4 ± 9.5	0.363

Values are shown as number (%) or mean ± SD

*ECMO* extracorporeal membrane oxygenation, *FiO*_*2*_ fraction of inspired oxygen saturation, *HCO*_*3*_ arterial bicarbonate level, *MAP* mean airway pressure, *PEEP* positive end-expiratory pressure, *PIP* peak inspiratory pressure, *PO*_*2*_ partial pressure of arterial oxygen, *SaO*_*2*_ arterial oxygen saturation

^a^The worst values in the previous 6 h were collected

### ECMO details

**Fig. 2:**
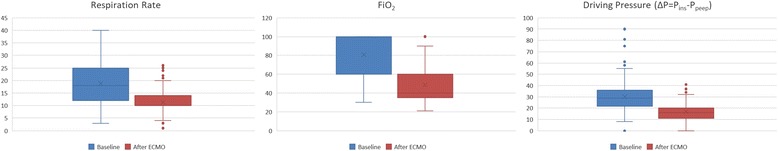
Difference in mechanical ventilator settings between pre-extracorporeal membrane oxygenation (baseline) and after extracorporeal membrane oxygenation initiation. The driving pressure (ΔP) significantly improved after ECMO initiation. ECMO, extracorporeal membrane oxygenation *p< 0.001

Venovenous ECMO was the most common mode used (93.9%, Table 2). Double lumen cannulation was used in 43.5% of patients. The mean duration of ECMO support was 176.4 hours, and 63.2% (163/258) of patients were weaned off of ECMO within 7 days. Improvement was characterized using the best ventilator settings occurring within 24 hours of ECMO initiation. Mean FiO_2_ was 48.8%; mean PIP was 25.0 cmH_2_O; the mean of the mean airway pressures was 14.2 cmH_2_O; and the average driving pressure was 16.8 cmH_2_O. Respiration rate, FiO_2_, PIP, and mean airway pressure significantly decreased within 24 hours of ECMO initiation (rate (breaths/min), 19.0 vs. 11.3, *p* < 0.001; FiO_2_ (%), 81.2 vs. 48.8, *p* < 0.001; PIP (cmH_2_O), 38.2 vs. 25.0, *p* < 0.001; mean airway pressure (cmH_2_O), 21.4 vs. 14.2, *p* < 0.001). In particular, driving pressure was significantly decreased after ECMO support (29.5 vs. 16.8 cmH_2_O, *p* < 0.001, Fig. 2).

**Table 2:** Clinical parameters related to mechanical ventilation and extracorporeal membrane oxygenation support

Variables	Total *N* = 272	Survivor *N* = 227	Non-survivor *N* = 45	*p*
ECMO mode^a^				
VV	131 (50.4)	110 (50.7)	21 (48.8)	0.824
VVDL	113 (43.5)	94 (43.3)	19 (44.2)	0.916
VV to VA	3 (1.2)	1 (0.5)	2 (4.7)	0.019
VA	5 (1.9)	5 (2.3)	0	0.315
VVDL + V	6 (2.3)	5 (2.3)	1 (2.3)	0.993
Other	2 (0.8)	2 (9.2)	0	0.527
Hours on ECMO	176.4 ± 192.7	161.6 ± 172.1	257.2 ± 268.4	0.035
Post-ECMO ventilator setting^b^				
Rate, breaths/min	11.3 ± 4.3	11.1 ± 4.2	12.7 ± 5.0	0.043
FiO_2_, %	48.8 ± 19.0	47.4 ± 17.8	57.4 ± 23.6	0.025
PIP, cmH_2_O	25.0 ± 7.8	24.3 ± 4.5	29.7 ± 8.3	< 0.001
PEEP, cmH_2_O	8.1 ± 4.5	7.9 ± 4.5	9.0 ± 4.0	0.195
MAP, cmH_2_O	14.2 ± 10.0	14.2 ± 10.4	14.6 ± 6.2	0.866
Driving pressure, cmH_2_O	16.8 ± 7.8	16.2 ± 7.8	21.1 ± 6.6	0.002

Values are shown as number (%) or mean ± SD

*ECMO* extracorporeal membrane oxygenation, *FiO*_*2*_ fraction of inspired oxygen saturation, *HCO*_*3*_- arterial bicarbonate level, *MAP* mean airway pressure, *PEEP* positive end-expiratory pressure, *PIP* peak inspiratory pressure, *PO*_*2*_ partial pressure of arterial oxygen, *SaO*_*2*_ arterial oxygen saturation, *VV* venovenous, *VVDL* venovenous double lumen, *VA* venoarterial, *VVDL + V* double lumen venovenous ECMO with a cephalic draining cannula

^a^Available in 260 patients

^b^The best values in the last 24 h were collected

### Complications and in-hospital mortality

In-hospital complications are shown in Table 3 and Additional file 2. Complications occurred in 177 patients (65.1%). Hemorrhagic complications were the most common (28.3%, 77/272). Other complications included renal (26.8%, 73/272), cardiovascular (26.1%, 71/272), mechanical (24.6%, 67/272), and metabolic (22.4%, 61/272) complications, culture-proven infection (16.5%, 45/272), neurologic complications (4.8%, 13/272), and limb ischemia (2.6%, 7/272). Among the hemorrhagic complications, cannulation-site hemorrhage was the most common (13.6%, 37/272). Other hemorrhagic complications included surgical-site hemorrhage (8.5%), pulmonary hemorrhage (5.1%) and gastrointestinal hemorrhage (2.6%). There were four deaths due to hemorrhage (1.5%).

**Table 3:** Complications and outcomes during extracorporeal membrane oxygenation support

Variables	Total *N* = 272	Survivor *N* = 227	Non-survivor *N* = 45	*p*
Mechanical	67 (24.6)	48 (21.1)	19 (42.2)	0.003
Oxygenator failure	14 (5.1)	7 (3.1)	7 (15.6)	0.001
Clots: oxygenator	31 (11.4)	26 (11.5)	5 (11.1)	0.947
Clots: other	10 (3.7)	6 (2.6)	4 (8.9)	0.042
Cannula problems	12 (4.4)	9 (4.0)	3 (6.7)	0.304
Total bleeding	77 (28.3)	52 (22.9)	25 (55.6)	< 0.001
GI hemorrhage	7 (2.6)	5 (2.2)	2 (4.4)	0.386
Cannulation site bleeding	37 (13.6)	25 (11.0)	12 (26.7)	0.005
Surgical site bleeding	23 (8.5)	16 (7.0)	7 (15.6)	0.061
Pulmonary hemorrhage	14 (5.1)	7 (3.1)	7 (15.6)	0.001
Neurologic	13 (4.8)	9 (4.0)	4 (8.9)	0.157
Brain death	6 (2.2)	3 (1.3)	3 (6.7)	0.026
Seizures	7 (2.6)	6 (2.6)	1 (2.2)	0.871
Cerebral infarction	8 (2.9)	2 (0.9)	6 (13.3)	< 0.001
Cerebral hemorrhage	12 (4.4)	7 (3.1)	5 (11.1)	0.017
Renal	73 (26.8)	55 (24.2)	18 (40)	0.029
Dialysis required	54 (19.9)	39 (17.2)	15 (33.3)	0.013
Cardiovascular	71 (26.1)	57 (25.1)	14 (31.1)	0.402
Infection culture proven	45 (16.5)	34 (15.0)	11 (24.4)	0.118
Pneumothorax requiring treatment	14 (5.1)	12 (5.3)	2 (4.4)	0.815
Metabolic	61 (22.4)	50 (22.0)	11 (24.4)	0.722
Limb ischemia	7 (2.6)	5 (2.2)	2 (4.4)	0.386
Total complication	177 (65.1)	138 (60.8)	39 (86.7)	0.001
Weaning success	234 (86.7)	227 (100)	7 (15.6)	< 0.001

Values are shown as number (%) or mean ± SD

*ECMO* extracorporeal membrane oxygenation, *GI* gastrointestinal

Overall, the rate of weaning success was 86.7% (234/272) and the rate of survival to discharge was 83.5% (227/272). Among non-survivors, 15.6% (7/45) died after ECMO weaning. The most common reason for death was organ failure (37.8%, 17/45; Additional file 2).

### Differences between survivors and non-survivors

Baseline ECMO patient profiles were significantly different between survivors and non-survivors (Table 1). The mean age was lower in survivors (34.7 vs. 43.4, *p* = 0.001), as was the mean pH (7.1 vs. 7.2, *p* = 0.045). Saturation was higher in survivors (92.3 vs. 85.2, *p* = 0.030). In addition, PEEP was lower in survivors (7.8 vs. 11.5, *p* = 0.002). ECMO duration and ventilator settings after ECMO initiation were significantly different between survivors and non-survivors (Table 2). The mean duration of ECMO was shorter in survivors (161.6 vs. 257.2, *p* = 0.035). The values of rate, FiO_2_, PIP, and driving pressure after ECMO initiation were lower in survivors (rate (breaths/min), 11.1 vs. 12.7, *p* = 0.043; FiO_2_ (%), 47.4 vs. 57.4, *p* = 0.025; PIP (cmH_2_O), 24.3 vs. 29.7, *p* < 0.001; driving pressure (cmH_2_O), 16.2 vs. 21.1, *p* = 0.002).

The complication rate was higher in non-survivors (60.8 vs. 86.7, *p* = 0.001; Table 3). The rate of mechanical complications was also higher in non-survivors (21.1 vs. 42.2, *p* = 0.003), especially the rate of oxygenator failure (3.1 vs. 15.6, *p* = 0.001) and clots (0.4 vs. 8.9, *p* = 0.042). The rate of hemorrhage in general (22.9 vs. 55.6, *p* = 0.001), the rate of cannulation-site hemorrhage (11.0 vs. 26.7, *p* = 0.005), and the rate of pulmonary hemorrhage (3.1 vs. 15.6, *p* = 0.001) were higher in non-survivors. Among neurologic complications, the rates of brain death (1.3 vs. 6.7, *p* = 0.026), cerebral infarction (0.9 vs. 13.3, *p* < 0.001), and cerebral hemorrhage (3.1 vs. 11.1, *p* = 0.017) were higher in non-survivors. In addition, renal complications occurred more often in non-survivors (24.2% vs. 40%, *p* = 0.029), especially the rate of dialysis (17.2 vs. 40, *p* = 0.013).

### Factors associated with in-hospital mortality

Univariate and multivariate analyses were performed to investigate the possible predictors of in-hospital mortality in the study population (Table 4). In the univariate analysis, age (odds ratio (OR), 1.05, 95% confidence interval (CI), 1.02–1.07, *p* < 0.001), mechanical complication (OR, 2.73, 95% CI, 1.39–5.34, *p* = 0.003), hemorrhage (OR, 4.21, 95% CI 2.16–8.18, *p* < 0.001), dialysis on ECMO (OR, 2.41, 95% CI, 1.19–4.90, *p* = 0.015), pre-ECMO PEEP (OR, 1.10, 95% CI, 1.03–1.18, *p* = 0.003), pre-ECMO pH (OR, 7.57, 95% CI, 1.04–55.29, *p* = 0.046), post-ECMO FiO_2_ (OR, 1.02, 95% CI, 1.01–1.04, *p* = 0.006), post-ECMO PIP (OR, 1.08, 95% CI, 1.03–1.14, *p* = 0.001), and post-ECMO driving pressure (OR, 1.08, 95% CI, 1.03–1.13, *p* = 0.003) were associated with increased in-hospital mortality. Among hemorrhagic complications, cannulation-site hemorrhage (OR, 2.94, 95% CI, 1.35–6.41, *p* = 0.007), central nervous system hemorrhage (OR, 3.93, 95% CI, 1.19–12.99, *p* = 0.025), and pulmonary hemorrhage (OR, 5.79, 95% CI, 1.92–17.44, *p* = 0.002) were associated with increased in-hospital mortality.

**Table 4:** Odds ratios of mortality at discharge

Variables	Univariate analysis		Multivariate analysis	
	OR (95% CI)	*p*	OR (95% CI)	*p*
Age	1.05 (1.02–1.07)	< 0.001	1.05 (1.01–1.08)	0.012
Mechanical complications	2.73 (1.39–5.34)	0.003		
Bleeding	4.21 (2.16–8.18)	< 0.001	2.97 (1.07–8.24)	0.036
Dialysis	2.41 (1.19–4.90)	0.015		
Pre-ECMO PEEP^a^, cmH_2_O	1.10 (1.03–1.18)	0.003	1.10 (1.01–1.20)	0.027
Pre-ECMO pH^a^	7.57 (1.04–55.29)	0.046		
Post-ECMO Fio2^b^, %	1.02 (1.01–1.04)	0.006	1.03 (1.00–1.05)	0.034
Post-ECMO PIP^b^, cmH_2_O	1.08 (1.03–1.14)	0.001		
Post-ECMO driving pressure^b^, cmH_2_O	1.08 (1.03–1.13)	0.003	1.08 (1.02–1.15)	0.011

*OR* Odds ratio, *CI* confidential interval, *ECMO* extracorporeal membrane oxygenation, *PEEP* positive end-expiratory pressure, *FiO*_*2*_ fraction of inspired oxygen saturation, *PIP* peak inspiratory pressure

^a^The worst values in the previous 6 h were collected

^b^The best values in the last 24 h were collected

In the multivariate logistic regression model, age (OR, 1.05, 95% CI, 1.01–1.08, *p* = 0.012), bleeding (OR, 2.97, 95% CI, 1.07–8.24, *p* = 0.036), pre-ECMO PEEP (OR, 1.10, 95% CI, 1.01–1.20, *p* = 0.027), post-ECMO FiO_2_ (O,: 1.03, 95% CI, 1.00–1.05, *p* = 0.034), and post-ECMO driving pressure (OR, 1.08, 95% CI, 1.02–1.15, *p* = 0.011) were associated with increased in-hospital mortality.

## Discussion

To our knowledge, this is the largest study of patients who have received ECMO for NFA refractory to mechanical ventilation. In this study, ventilator settings significantly improved after ECMO initiation to allow protective ventilation. Overall survival to hospital discharge was 83.5%, which was favorable compared to the ECMO outcome of other types of respiratory failure. Otherwise, ECMO complication was still common, even though it was mostly not serious. The total complication rate was 65.1%, with hemorrhagic complications being the most common (28.3%). Although bleeding was associated with increased in-hospital mortality, fatal bleeding only occurred in 1.5% of cases and most other complications were not fatal. Therefore, ECMO may be an effective treatment to prevent ventilator-induced lung injury in NFA refractory to mechanical ventilation. In addition, careful monitoring is required to detect and manage complications early and reduce their impact.

The mortality rate of patients with asthma is decreasing, but remains significant [9, 10]. While mechanical ventilation is a potentially life-saving intervention, a large proportion of the morbidity and mortality seen in patients with asthma may be related to the mechanical ventilation itself rather than to disease progression [15–18]. Regardless of the mode of ventilation selected, mechanical ventilation in NFA should aim to avoid barotrauma, minimize dynamic hyperinflation, maintain adequate oxygenation, and allow some degree of permissive hypercapnia until bronchodilators and steroids improve airflow [19]. However, this strategy may be impossible in patients with severe NFA. A new therapeutic approach is needed to reduce lung damage in patients with severe NFA requiring treatment with maximal ventilator settings.

Traditionally, overt hypercapnic acidosis in patients with acute severe asthma is significantly associated with higher rates of invasive ventilation; this leads to longer hospital stays, more complications, and a higher mortality rate [20]. In general, it is recommended that a moderate degree of hypercarbia and respiratory acidosis be tolerated in NFA, and that airway pressure be limited by using low respiratory rates and low tidal volumes [21]. Severe hypercapnea can recover rapidly once effective treatment has begun and hypoxemia is corrected. However, in severe cases with extreme airway obstruction, hypoxemia cannot be corrected despite maximal use of the mechanical ventilator. Death can occur as a result of asphyxia due to extreme airflow limitation and resulting hypoxia.

Despite limited data about physiologic and ventilator parameters, ventilator settings after ECMO initiation significantly improved, particularly driving pressure and PIP. Driving pressure is considered to be a reasonable surrogate for transpulmonary pressure, and high transpulmonary pressure can cause lung injury or gross barotrauma [22, 23]. The high PIP seen before ECMO initiation may reflect possible dynamic hyperinflation, which can result in cardiovascular instability and barotrauma to the lung. Our results suggest that ECMO significantly improves gas exchange, despite a lower PIP and lower driving pressure. Therefore, the use of ECMO in NFA may reduce ventilator-induced lung injury and oxygen toxicity by allowing for decreased ventilator settings. In NFA, the time to restoration of airway patency and reactivity following conventional treatment is highly variable, and clinicians cannot predict when bronchospasm will resolve. ECMO should be considered before refractory NFA causes barotrauma and volutrauma. In particular, the ventilation/perfusion mismatch produced by increased airway resistance and airflow limitation may facilitate severe hypoxemia in NFA. Therefore, ECMO could be considered in patients with persistent signs of deterioration including severe hypoxemia, acidosis, and hemodynamic instability, despite maximal mechanical ventilation settings.

Complications were common, even though the rates were lower than in previous studies [9]. However, the total complication rate included all minor complications, even those unrelated to ECMO, such as hyperglycemia. Hemorrhage was the most common complication, particularly at the cannulation site. In general, cannulation-site hemorrhage is less lethal, and easier to manage than bleeding at other sites. Overall, there were few fatal complications and only four patients died due to hemorrhage (1.5%, Additional file 2). The hemorrhagic complications of ECMO support are known to significantly impact patient survival and quality of life [24]. In this study, hemorrhage was independently associated with increased in-hospital mortality. Maintaining the balance between hemostasis and anticoagulation is difficult, and it remains a major challenge during ECMO [25, 26]. Unfortunately, the ELSO Registry did not include information about anticoagulation management. Clinicians should assess patient risk factors for ECMO-related hemorrhage, and actively take steps to correct them. In selected cases, termination of ECMO support may be required to prevent hemorrhage. Fortunately, ECMO stabilzes platelet function and the risk of hemorrhage is low in the early period of administration [27]. Furthermore, there is a recent trend toward conservative anticoagulation strategies to decrease complications due to hemorrhage [24, 28]. Considering the reversibility of airflow obstruction in asthma, ECMO can be safely used in these patients for a short period of time. However, careful management is required to balance the risks and benefits. The need for ECMO support should be reassessed when hemorrhagic complications are likely to be fatal.

This study had several limitations. First, we analyzed data voluntarily submitted by various international centers; these data may be susceptible to information bias, reporting bias, and uncontrolled confounding factors. Second, we defined patients with NFA as those with a primary ICD-9 diagnosis code of asthma. This may limit the applicability of these results to certain cases. Third, the ABGA values following ECMO initiation were not always present in this dataset, and as such their improvement could not be assessed as a secondary outcome. Furthermore, many of the baseline ventilator settings in this dataset did not adhere to the generally recommended criteria [21]. In these cases, refractory airway obstruction and unstable blood gas profiles might be related to suboptimal ventilator strategies. While this registry may not be representative of all cases, the strength of this study was that cases were extracted from a relatively large, international dataset. The data are also contemporary and reflect the current status of ECMO use in NFA. A further strength of the study was that the detailed complication profiles are present in the ELSO data. Because asthma is a reversible disease, it is important to know the complications associated with therapeutic interventions. This analysis provides important and current information to guide a rapid increase in the use of ECMO in NFA.

## Conclusions

The management of NFA remains a significant problem in critical care. Mechanical ventilation of patients with NFA is challenging, and high ventilator settings may cause lung injury and hemodynamic instability secondary to barotrauma and dynamic hyperinflation. As NFA is ultimately reversible, clinicians should actively consider ECMO to both provide adequate gas exchange and prevent lung injury. In this study, ECMO provided full respiratory support in patients with NFA, and its use resulted in acceptable survival to hospital discharge. However, ECMO-related complications are common. To encourage the use of ECMO as a rescue therapy, understanding and reducing ECMO-related complications should be a priority. The use of ECMO to reduce ventilator-induced lung injury may reduce overall NFA mortality worldwide. Further clinical research into the use of ECMO in this context is needed.

## Additional files

Additional file 1: Figure S1. This graph shows the increasing trend in extracorporeal membrane oxygenation use in adults with near-fatal asthma. (TIF 80 kb)

Additional file 2: Table S1. Complications. **Table S2.** Reason for extracorporeal membrane oxygenation discontinuation and mortality. (DOC 80 kb)

## Abbreviations

ABGA: Arterial blood gas analysis
ECMO: Extracorporeal membrane oxygenation
ECPR: Extracorporeal cardiopulmonary resuscitation
ELSO: Extracorporeal Life Support Organization
FiO_2_: Fraction of inspired oxygen
ICD-9: International Classification of Diseases 9th edition
NFA: Near-fatal asthma
PEEP: Positive end-expiratory pressure
PIP: Peak inspiratory pressure
VV: Venovenous
