# Supplementary figures and images for: Extracorporeal membrane oxygenation for life-threatening asthma refractory to mechanical ventilation: analysis of the Extracorporeal Life Support Organization registry
Source: Crit Care. 2017 Dec 6;21:297. doi: 10.1186/s13054-017-1886-8 (PMC5719729; doi:10.1186/s13054-017-1886-8)

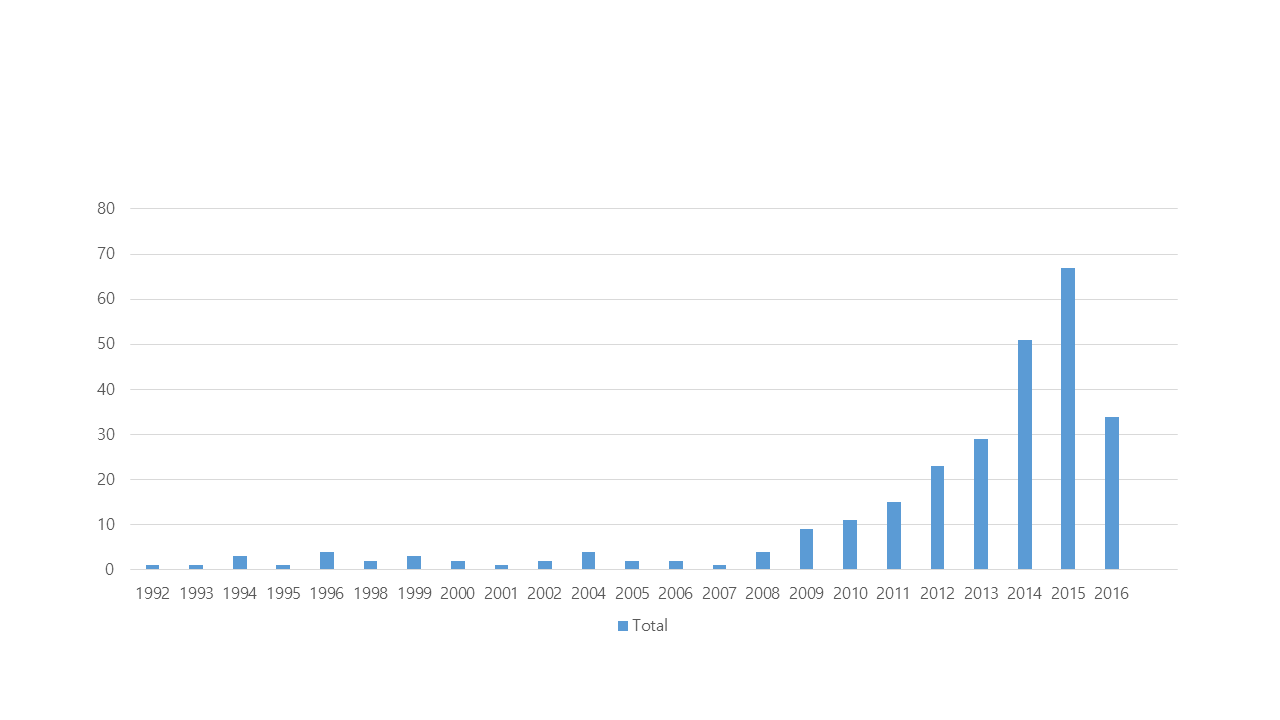

Supplement: Supplementary file 1 — This graph shows the increasing trend in extracorporeal membrane oxygenation use in adults with near-fatal asthma. (TIF 80 kb) [file 13054_2017_1886_MOESM1_ESM.tif]
