# Supplementary material for: Extracorporeal membrane oxygenation for life-threatening asthma refractory to mechanical ventilation: analysis of the Extracorporeal Life Support Organization registry
Source: Crit Care. 2017 Dec 6;21:297. doi: 10.1186/s13054-017-1886-8 (PMC5719729; doi:10.1186/s13054-017-1886-8)
Supplement: Supplementary file 2 — Complications. Table S2. Reason for extracorporeal membrane oxygenation discontinuation and mortality. (DOC 80 kb) [file 13054_2017_1886_MOESM2_ESM.doc]

Table S1. Complications

| Variables | Total  N=272 | Survivor  N=227 | Non-survivor  N=45 | *P* |
| --- | --- | --- | --- | --- |
| Mechanical | 67 (24.6) | 48 (21.1) | 19 (42.2) | 0.003 |
| Oxygenator failure | 14 (5.1) | 7 (3.1) | 7 (15.6) | 0.001 |
| Clots: oxygenator | 31 (11.4) | 26 (11.5) | 5 (11.1) | 0.947 |
| Clots: other | 10 (3.7) | 6 (2.6) | 4 (8.9) | 0.042 |
| Cannular problems | 12 (4.4) | 9 (4.0) | 3 (6.7) | 0.304 |
| Total bleeding | 77 (28.3) | 52 (22.9) | 25 (55.6) | <0.001 |
| GI hemorrhage | 7 (2.6) | 5 (2.2) | 2 (4.4) | 0.386 |
| Cannulation site bleeding | 37 (13.6) | 25 (11.0) | 12 (26.7) | 0.005 |
| Surgical site bleeding | 23 (8.5) | 16 (7.0) | 7 (15.6) | 0.061 |
| Pulmonary hemorrhage | 14 (5.1) | 7 (3.1) | 7 (15.6) | 0.001 |
| Hemolysis | 6 (2.2) | 5 (2.2) | 1 (2.2) | 0.993 |
| DIC | 7 (2.6) | 5 (2.2) | 2 (4.4) | 0.386 |
| Neurologic | 13 (4.8) | 9 (4.0) | 4 (8.9) | 0.157 |
| Brain death | 6 (2.2) | 3 (1.3) | 3 (6.7) | 0.026 |
| Seizures | 7 (2.6) | 6 (2.6) | 1 (2.2) | 0.871 |
| Cerebral infarction | 8 (2.9) | 2 (0.9) | 6 (13.3) | <0.001 |
| Cerebral hemorrhage | 12 (4.4) | 7 (3.1) | 5 (11.1) | 0.017 |
| Renal | 73 (26.8) | 55 (24.2) | 18 (40) | 0.029 |
| Creatinine 1.5-30 mg/dL | 27 (9.9) | 21 (9.3) | 6 (13.3) | 0.403 |
| Creatinine >3.0 mg/dL | 20 (7.4) | 14 (6.2) | 6 (13.3) | 0.092 |
| Dialysis required | 54 (19.9) | 39 (17.2) | 15 (33.3) | 0.013 |
| Cardiovascular | 71 (26.1) | 57 (25.1) | 14 (31.1) | 0.402 |
| CPR required | 7 (2.6) | 5 (2.2) | 2 (4.4) | 0.386 |
| Inotropes on ECMO | 69 (25.4) | 56 (24.7) | 13 (28.9) | 0.552 |
| Tamponade : blood | 3 (1.1) | 3 (1.3) | 0 | 0.438 |
| Infection culture proven | 45 (16.5) | 34 (15.0) | 11 (24.4) | 0.118 |
| Pneumothorax requiring treatment | 14 (5.1) | 12 (5.3) | 2 (4.4) | 0.815 |
| Metabolic | 61 (22.4) | 50 (22.0) | 11 (24.4) | 0.722 |
| Hypoglycemia <40mg/dL | 3 (1.1) | 2 (0.9) | 1 (2.2) | 0.431 |
| Hyperglycemia >240mg/dL | 20 (7.4) | 17 (7.5) | 3 (6.7) | 0.847 |
| pH<7.20 | 33 (12.1) | 28 (12.3) | 5 (11.1) | 0.818 |
| pH>7.60 | 8 (2.9) | 7 (3.1) | 1 (2.2) | 0.755 |
| Hyperbilirubinemia | 19 (7.0) | 15 (6.6) | 4 (8.9) | 0.583 |
| Limb ischemia | 7 (2.6) | 5 (2.2) | 2 (4.4) | 0.386 |
| Total complication | 177 (65.1) | 138 (60.8) | 39 (86.7) | 0.001 |
| Weaning success | 234 (86.7) | 227 (100) | 7 (15.6) | <0.001 |

Values are shown as number (%) or mean ± SD.

Hemolysis: plasma-free hemoglobin > 50 mg/dL

Hyperbilirubinemia: direct > 2 mg/dL, indirect > 13 mg/dL, or total > 15 mg/dL

ECMO: extracorporeal membrane oxygenation, DIC: disseminated intravascular coagulation

Table S2. Reason for ECMO discontinuation and mortality

| Reason | Non-survivors (N=45) |
| --- | --- |
| Died for family request | 4/45 (8.9) |
| Died for hemorrhage | 4/45 (8.9) |
| Diagnosis incompatible with life | 8/45 (17.8) |
| Died for organ failure | 17/45 (37.8) |
| Reason not specified | 5/45 (11.1) |
| Weaned but later died | 7/45 (15.6) |

Values are shown as number (%) or mean ± SD.

ECMO: extracorporeal membrane oxygenation
